# Morphometric diagnosis of *Glossina palpalis* (Diptera: Glossinidae) population structure in Ghana

**DOI:** 10.1186/s13104-017-3113-8

**Published:** 2017-12-29

**Authors:** Faith Ebhodaghe, Maxwell Kelvin Billah, Delphina Adabie-Gomez, Adam Yahaya

**Affiliations:** 1 African Regional Postgraduate Programme in Insect Science, West-African Sub-Regional Centre, University of Ghana Legon, Accra, Ghana; 2 Department of Animal Biology and Conservation Science, University of Ghana Legon, Accra, Ghana; 3 Tsetse and Trypanosomiasis Control Unit/PATTEC, Ministry of Food and Agriculture, Pong-Tamale, Ghana

**Keywords:** Ghana, *Glossina palpalis*, Population

## Abstract

**Objective:**

This study aimed to identify isolated population(s) of *Glossina palpalis* in Ghana using geometric morphometrics to evaluate variations in wing-shape and size between populations of the fly from three regions.

**Results:**

Wing shape of *G. palpalis* tsetse flies from the Northern, Western and Eastern Regions varied significantly between each other. Populations from the Northern and Western Regions varied the most (Mahalanobis Distance = 54.20). The least variation was noticed between populations from the Western and Eastern Regions (MD = 1.99). On morphospace, the Northern population clearly separated from the Eastern and Western populations both of which overlapped. Wing centroid size also significantly varied among populations. Reclassification scores were satisfactory reaching 100% for the Northern population. The Northern population of *G. palpalis* is possibly isolated from the Western and Eastern Region populations. Meanwhile, a panmictic relationship could be on-going between the Western and Eastern populations. We speculate that geographical distance and subspecific difference between populations are among factors responsible for observed pattern of wing shape variations among the studied populations. The implications of results regarding choice of control strategy and limitations of the study are discussed.

## Introduction

African trypanosomiasis is a disease of man and livestock in Sub-Saharan Africa. The disease constrains human and animal health, and retards socioeconomic development [1–3]. Infection with trypanosomes, the etiological agents of African trypanosomiasis, results through infective bites by tsetse flies during bloodmeals. To eliminate the problem of African trypanosomiasis, present control efforts are partly geared towards eradicating the tsetse fly vectors [4].

Tsetse eradication is more easily achievable on Islands than on mainlands. On Islands, tsetse populations are isolated and as a result can be readily targeted for elimination and eradication [5, 6]. But on mainlands, populations are commonly panmictic. Consequently, re-infestation could result, that is, in a circumstance where only a subpopulation of an entire panmictic population was controlled [4, 7, 8]. However, in some cases, due to fragmentation of tsetse habitats by anthropogenic activities, climate differences etc., it is possible for tsetse populations to become isolated on mainlands. Thus, to reduce tsetse re-infestations on mainlands and therefore fast-track their eradication, attempts are now being made to identify such isolated populations [9–12].

In Ghana, the human form of African trypanosomiasis was last reported in 2013 [13]. But the animal trypanosomiasis remains highly endemic and *Glossina palpalis*, which is perhaps the most spatially distributed tsetse fly species within the country, is the primary vector of the disease [14–16]. Insect wing morphometric properties are influenced by genes and, as a result, may provide information on tsetse population structure [7, 9, 11, 12]. Wing shape was used to investigate *G. palpalis palpalis* population structure in Ivory Coast [9]. In the study, seventy-nine percent (79%) of wing shape variations between populations of the tsetse fly was explained by genetic variations after microsatellite DNA analysis. Wing morphometry also favourably compared with microsatellite DNA analysis in Guinea where the Loos Island *G. p. gambiensis* population was reportedly isolated from the mainland [11].

We therefore conducted a study to diagnose *G. palpalis* population structure for presence of isolated population(s) using the cost effective geometric morphometrics technique to assess for variations in wing morphometry (shape and size) among populations from three regions, namely, Northern, Western and Eastern Regions. The regions were selected on the basis of their location in different parts of the country which could present a clearer picture of *G. palpalis* population structure in Ghana than when an area of limited spatial coverage is considered.

## Main text

### Tsetse fly populations and study areas

**Fig. 1:**
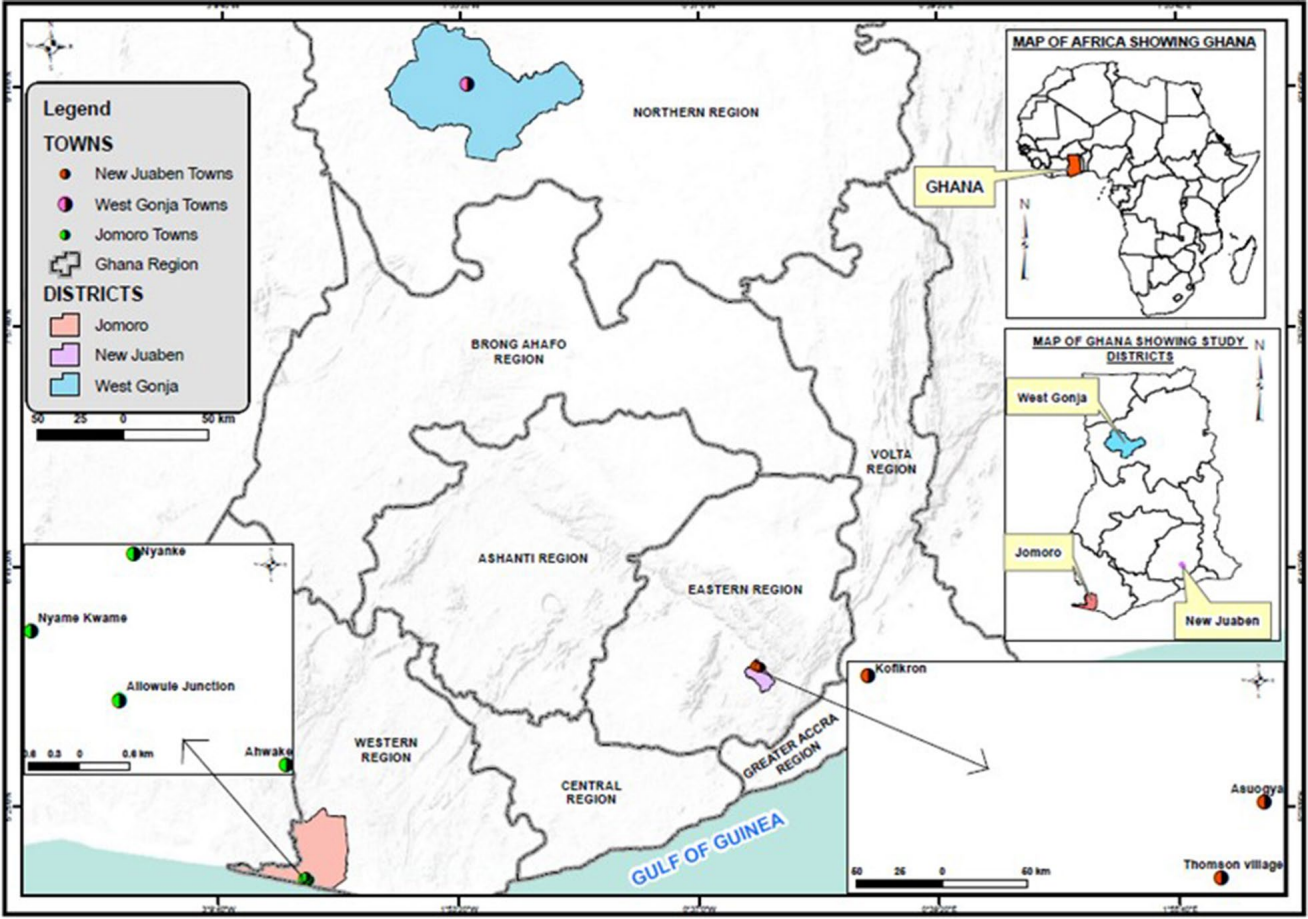
Map of Ghana showing areas where tsetse flies were collected for geometric morphometric analysis. Map was designed by Ms. Nana Amah of the Remote Sensing and Geographic Information System Laboratory, University of Ghana Legon, Accra, Ghana

The study populations were from the Northern and Southern sectors of Ghana. In the Northern sector, tsetse samples were collected from the Mole National Game Reserve in the Northern Region, while in the Southern sector, samples were from tsetse-endemic locations in the Eastern and Western Regions (Table 1; Fig. 1). The three regions occupy different ecological zones: Western Region is in the tropical rainforest zone; Eastern Region in the semi-deciduous rain forest zone; and the Northern Region in the savannah grassland. From the Southern to Northern Sector, temperature ranges from 18 °C to over 40 °C, and annual rainfall from 2000 to 1100 mm [17].

**Table 1:** Study areas where tsetse fly samples were collected and number of tsetse flies used for analysis

Ecological Zone	Region	Collection site	Coordinate		Altitude (m)	No. collected	No. analyzed
			Latitude (N)	Longitude (W)			
Tropical rainforest	Western	Ahwake	5°1′52″	2°41′5″	3	436	35
		Allowule junction	5°2′17″	2°42′10″	20	234	35
		Nyame Kwame	5°2′44″	2°42′44″	32	52	15
		Nyanke	5°3′14″	2°42′4″	7	95	8
Semi-deciduous rainforest	Eastern	Asuogya	6°10′2″	0°17′34″	204	22	8
		Kofikron	6°10′29″	0°18′57″	190	68	39
		Thomson village	6°9′46″	0°17′43″	192	15	6
Savannah grassland	Northern	Mole reserve	9°17′49″	1°46′20″	125	49	29

### Collection of tsetse fly samples

Tsetse flies were sampled in August to October, 2016. Biconical traps without attractants were used for the collection. We located traps within livestock producing areas in the Eastern and Western Regions, but along riparian vegetation in the Northern Region. Collected tsetse flies were harvested from traps after every 24 h, while *G. palpalis* were morphologically identified [18], sorted into sexes, and stored in 70% alcohol within small labeled vials until morphometric analysis.

### Preparation of tsetse fly wings

A total of 175 *G. palpalis* was analyzed using geometric morphometrics: 93, 53 and 29 flies from the Western, Eastern and Northern Regions, respectively (Table 1). Variations in number of flies analyzed for each region were influenced by number of flies collected and those whose wings remained intact and undamaged at the point of analysis. We considered only the right wings of female tsetse flies for analyses to avoid asymmetric and sexual dimorphic effects [19, 20].

Wings were detached from each fly and mounted in glycerol between glass slides and cover slips. Wing images were photographed at 12.5× magnification using an EZ4D microscope with an inbuilt camera connected to a laptop computer where captured images were archived.

### Data acquisition

One individual collected 11 landmark coordinates on each wing image [21]. Landmarks were collected in the same order for each wing. Digitized image not displayed. See section on ‘availability of data and materials’ below.

### Geometric morphometric analysis

#### Wing shape

Raw landmark coordinates were transformed and superimposed by the Generalised Proscrustes method to generate shape variables by removing effects of scale, location, and orientation [22, 23]. Shape variables in the form of partial warps scores were subjected to discriminant analysis to produce Mahalanobis distances used to quantify wing shape variations between *G. palpalis* populations. Mahalanobis distances were tested for significant differences by the non-parametric method at 1000 permutations with Bonferroni’s correction. *p* value was significant at < 0.05.

Morphospace was generated based on the first two canonical variates for graphical depiction of wing shape variations.

A reclassification test was performed to verify the accuracy of the geometric morphometric technique to correctly diagnose *G. palpalis* populations [24].

#### Wing size

Geometric morphometrics allows derivation of centroid sizes from landmark coordinates [25]. Centroid size is the square root of the sum of the squared distances between the center of the configuration of landmarks and each separate landmark. Significant difference in centroid sizes between populations was tested by the Kruskal–Wallis test, while the Wilcoxon-rank-sum test was used for pairwise comparisons with Bonferroni *p* value adjustment method at significant level of p < 0.05.

#### Softwares

Analyses were conducted in different modules of the CLIC package [23] and in R software (version 3.3.2).

### Results

#### Wing shape

Mahalanobis Distances (MDs) were significantly different (*p* < 0.01667) between *G. palpalis* populations from the three study regions. The distance was highest between populations from the Northern and Western Regions (MD: 54.20), followed by Eastern and Northern Regions (MD: 53.44), and then Western and Eastern Regions (MD: 1.99). In each case, *p*-value was 0.000.

**Fig. 2:**
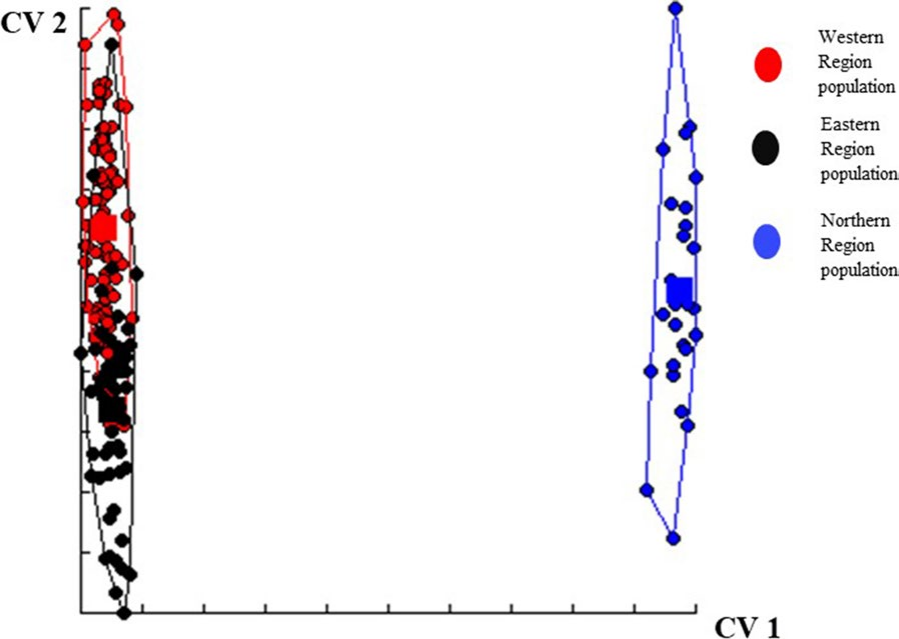
Morphospace showing wing shape variation pattern of *G. palpalis* populations from Northern, Western, and Eastern Regions of Ghana. Morphospace is based on the first two Canonical Variates from discriminant analysis of wing shape variables. Convex hulls delimit extent of wing shape variations within population. Circular points represent individual tsetse flies. Tsetse flies of the same population are represented by same colour points. Squares within hulls represent average shape of a population. Overlapping of hulls representing populations from the Western (red) and Eastern (black) Regions indicates similarity in wing shape. Separation of hull representing population from the Northern Region (blue) signifies its dis-similarity in wing shape from the other populations

Wing shape variations between populations were visualized on morphospace with the Northern population strongly separated from the other two populations (Fig. 2). However, Eastern and Western populations overlapped.

Reclassification scores were satisfactory, being highest for the Northern population (100%). Reclassification scores for the Western and Eastern populations were 81 and 83%, respectively.

#### Wing size

Overall mean centroid size was 568 mm. Western population had the greatest centroid size of 590.33 mm, followed by Eastern and Northern populations with centroid sizes of 567.90 and 496.97 mm, respectively. *Kruskal*–*Wallis* test was significant (Chi squared value: 108.88. p < 2.2x10−16). Similarly, Wilcoxon-rank-sum tests between each pair of populations were also significant (*p* values: West vs. North: < 2x10−16. East vs. West: 1.8x10−14. North vs. West: < 2x10−16).

### Discussion

The Northern population of *G. palpalis* was clearly isolated from other populations and thus can be targeted for elimination with little or no fear of re-infestation by populations from the Western or Eastern Regions. We also observed the Western and Eastern populations to be significantly different although, on morphospace, both populations overlapped suggesting panmixia. An attempt to sequentially control populations of the flies from these two regions may thus result in re-infestation. Therefore, both populations should be simultaneously controlled.

Geographical distance between the populations may have influenced observed pattern of segregation between populations [9]. The Northern and Western populations which were geographically farthest apart had the highest Mahalanobis distance, while the Western and Eastern populations which were closest had the least Mahalanobis distance. Similarly, in Ivory Coast, populations of *G. p. palpalis* within Abidjan when compared had low Mahalanobis distances [9]. Mahalanobis distances however increased when the Abidjan populations were compared to a population of the fly in Aniassue, an area of about 186 km away from Abidjan. Here, the locations of the study populations in the Western and Eastern Regions were 670 and 594 km respectively away from the Mole Game Reserve where tsetse were collected in the Northern Region. These long geographical distances from the Northern Region may have constrained exchange of genes between population from the Region and those from the Western and Eastern Regions.

More importantly, it is possible that the subspecific status of the populations contributed to their segregation. In Ghana, *G. palpalis* exists in two subspecific forms and these are *Glossina palpalis palpalis* in the rainforest zone [14, 16] and *G. p. gambiensis* in the savannah zone [26, 27]. In view of this, we assumed that population of *G. palpalis* from the Northern Region belonged to the *G. p. gambiensis* subspecies for which cause, it clearly separated from the Western and Eastern populations which are presumably *G. p. palpalis.* Population segregation may have been further strengthened by variability in climatic conditions between the regions to which each population has become adapted [28, 29].

Centroid size was significantly higher in the Western than Eastern and Northern Regions. Temperature and relative humidity influence pupae sizes during development in the soil. Large tsetse individuals result from pupae which developed in low temperature soils, and the reverse in soils with high temperatures [9]. The low temperature and high humidity in the Western Region may have therefore caused the larger sizes of tsetse flies from the area. This idea is also expressed in a previous study [9] and agrees with the Bergmann’s ecogeographical rule which states that organisms in low temperature areas have larger body sizes than their counterparts from high temperature locations.

Although wing shape and size were both significantly different between populations, the authors however recommend the use of wing shape in studies of tsetse population structure since it is more stable than size which could be easily influenced by changes in environmental variables [23].

The satisfactory reclassification scores obtained for each population indicates that geometric morphometrics which is cost effective could be an effective alternative, or more correctly, complementary approach to the molecular technique for diagnoses of tsetse population structure in Ghana [24, 30].

### Conclusion

In the present study, we found a strong structuring of *G. palpalis* populations between the Northern and Southern sectors of Ghana but a likely ongoing panmixia between populations from the Eastern and Western Regions. Geographical distances between populations, subspecific differences, and non-uniform climatic conditions may be responsible for the observed structuring between populations. Whatever the case, our data support adoption of sequential control of *G. palpalis* populations between the Northern and Southern Sectors without fear of re-infestation, but simultaneous control in Eastern and Western Regions. Finally, considering the high reclassification scores obtained in this study and documented favourable comparison of geometric morphometrics with molecular tools, we recommend geometric morphometric study of wing shape as an efficient technique for tsetse population study in Ghana.

### Limitations

A major limitation to this study was the non-utilization of molecular tools acclaimed to possess higher sensitivity than geometric morphometrics in tsetse population studies. Our non-utilization of molecular tools was due to unavailability of funds. Also, populations from the other five and two regions in southern and northern Ghana, respectively were not considered. Tsetse sampling activities are a costly endeavor thus we could only collect samples from three of the ten Regions of Ghana. Hopefully, these limitations would be addressed in the future pending availability of funds.

## Abbreviations

CLIC: collecting landmarks for identification and characterization
DNA: deoxyribonucleic acid
MD: Mahalanobis Distance
PATTEC: Pan African Tsetse and Trypanosomiasis Eradication Campaign
